# Improving species status assessments under the U.S. Endangered Species Act and implications for multispecies conservation challenges worldwide

**DOI:** 10.1111/cobi.13777

**Published:** 2021-06-28

**Authors:** Reed F. Noss, Jennifer M. Cartwright, Dwayne Estes, Theo Witsell, Gregg Elliott, Daniel Adams, Matthew Albrecht, Ryan Boyles, Patrick Comer, Chris Doffitt, Don Faber‐Langendoen, JoVonn Hill, William C. Hunter, Wesley M. Knapp, Michael E. Marshall, Jason Singhurst, Christopher Tracey, Jeffrey Walck, Alan Weakley

**Affiliations:** ^1^ Florida Institute for Conservation Science and Southeastern Grasslands Initiative Melrose Florida USA; ^2^ U.S. Geological Survey Lower Mississippi‐Gulf Water Science Center Nashville Tennessee USA; ^3^ Southeastern Grasslands Initiative Austin Peay State University Clarksville Tennessee USA; ^4^ Southeastern Grasslands Initiative Austin Peay State University, K Gregg Consulting Clarksville Tennessee USA; ^5^ U.S. Fish and Wildlife Service Cookeville Tennessee USA; ^6^ Center for Conservation and Sustainable Development Missouri Botanical Garden St. Louis Missouri USA; ^7^ U.S. Geological Survey Southeast Climate Adaptation Science Center Raleigh North Carolina USA; ^8^ NatureServe Boulder Colorado USA; ^9^ Natural Areas Registry Louisiana Department of Wildlife & Fisheries Pineville Louisiana USA; ^10^ NatureServe Syracuse New York USA; ^11^ Mississippi Entomological Museum Mississippi State University Starkville Mississippi USA; ^12^ U.S. Fish and Wildlife Service National Wildlife Refuge System Atlanta Georgia USA; ^13^ North Carolina Natural Heritage Program Asheville North Carolina USA; ^14^ Texas A&M University Natural Resource Institute Dallas Texas USA; ^15^ Texas Parks and Wildlife Department Nongame and Rare Species Program Austin Texas USA; ^16^ Pennsylvania Natural Heritage Program Pittsburgh Pennsylvania USA; ^17^ Department of Biology Middle Tennessee State University Murfreesboro Tennessee USA; ^18^ North Carolina Botanical Garden University of North Carolina, and Southeastern Grasslands Initiative Chapel Hill North Carolina USA

**Keywords:** ecosystem conservation, endemic species, grasslands, multispecies planning, southeastern United States, conservación del ecosistema, especie endémica, pastizales, planeación multiespecie, sureste de los Estados Unidos, 多物种规划, 生态系统保护, 美国东南部, 草地, 特有物种

## Abstract

Despite its successes, the U.S. Endangered Species Act (ESA) has proven challenging to implement due to funding limitations, workload backlog, and other problems. As threats to species survival intensify and as more species come under threat, the need for the ESA and similar conservation laws and policies in other countries to function efficiently has grown. Attempts by the U.S. Fish and Wildlife Service (USFWS) to streamline ESA decisions include multispecies recovery plans and habitat conservation plans. We address species status assessment (SSA), a USFWS process to inform ESA decisions from listing to recovery, within the context of multispecies and ecosystem planning. Although existing SSAs have a single‐species focus, ecosystem‐based research can efficiently inform multiple SSAs within a region and provide a foundation for transition to multispecies SSAs in the future. We considered at‐risk grassland species and ecosystems within the southeastern United States, where a disproportionate number of rare and endemic species are associated with grasslands. To initiate our ecosystem‐based approach, we used a combined literature‐based and structured World Café workshop format to identify science needs for SSAs. Discussions concentrated on 5 categories of threats to grassland species and ecosystems, consistent with recommendations to make shared threats a focus of planning under the ESA: (1) habitat loss, fragmentation, and disruption of functional connectivity; (2) climate change; (3) altered disturbance regimes; (4) invasive species; and (5) localized impacts. For each threat, workshop participants identified science and information needs, including database availability, research priorities, and modeling and mapping needs. Grouping species by habitat and shared threats can make the SSA process and other planning processes for conservation of at‐risk species worldwide more efficient and useful. We found a combination of literature review and structured discussion effective for identifying the scientific information and analysis needed to support the development of multiple SSAs.

*Article impact statement*: Species status assessments can be improved by an ecosystem‐based approach that groups imperiled species by shared habitats and threats.

## Abstract (es)

Mejoramiento de la Evaluación del Estado de una Especie bajo el Acta de Especies en Peligro de los Estados Unidos y Sus Consecuencias para los Retos de la Conservación Multiespecie a Nivel Mundial

**Resumen:**

A pesar de su éxito, el Acta de Especies en Peligro de los E.U.A. (AEP) ha sido un reto de implementación por las limitaciones en su financiamiento, el retraso en la carga de trabajo y otros problemas. Conforme se intensifican las amenazas a la supervivencia de las especies y más especies resultan amenazadas, aumenta la necesidad de que la AEP y las políticas similares de otros países funcionen efectivamente. Los intentos por parte del Servicio Estadounidense de Pesca y Fauna (SEPF) para optimizar las decisiones de la AEP incluyen planes multiespecie de recuperación y planes de conservación de hábitat (PRH). Abordamos la evaluación del estado de las especies (EEE), un proceso del SEPF para orientar las decisiones del AEP desde el listado hasta la recuperación, dentro del contexto de la planeación multiespecie y de ecosistemas. Aunque las EEE existentes tienen un enfoque sobre una única especie, la investigación basada en el ecosistema puede orientar eficientemente a múltiples EEE dentro de una región y proporcionar una base para la transición a las EEE multiespecie en el futuro. Consideramos a las especies y los ecosistemas en riesgo de los pastizales del sureste de los Estados Unidos, en donde un número desproporcionado de especies raras y endémicas está asociado con los pastizales. Para iniciar nuestra estrategia basada en el ecosistema, usamos un formato de taller de World Café estructurado y basado en la literatura para identificar la necesidad de tener EEE. Las discusiones se centraron en cinco categorías de amenazas para las especies y ecosistemas de los pastizales, consistentes con las recomendaciones para volver a las amenazas compartidas un foco de la planeación bajo la AEP: (1) pérdida del hábitat, fragmentación y disrupción de la conectividad funcional; (2) cambio climático; (3) regímenes alterados de perturbación; (4) especies invasoras; y (5) impactos localizados. Para cada amenaza, los participantes del taller identificaron las necesidades científicas y de información, incluyendo la disponibilidad de bases de datos, prioridades de la investigación y necesidades de modelado y mapeado. La agrupación de las especies por hábitat y amenaza compartida puede hacer más eficientes y útiles el proceso de EEE y otros procesos de planeación de la conservación de especies en riesgo a nivel mundial. Encontramos una combinación de revisiones bibliográficas y discusiones estructuradas para identificar la información y el análisis necesarios para respaldar el desarrollo de múltiples EEE.

## 【摘要】

尽管《美国濒危物种法案》 (U.S. Endangered Species Act, ESA) 已取得一定成功, 但由于资金限制、工作量积压及其它问题, 该法案的实施仍面临挑战。随着威胁的加剧和受到威胁的物种增加, 人们对于 ESA 及其它国家类似的保护法律和政策发挥有效作用的需求也越来越强烈。同时, 美国鱼类及野生动物管理局也在努力简化包括多物种恢复计划和栖息地保护计划在内的ESA决策过程。本研究以多物种和生态系统规划为背景, 进行了物种状态评估, 这是美国鱼类及野生动物管理局为物种从列入名录到恢复的一系列ESA决策提供信息的过程。现有的物种状态评估侧重于单一物种, 而基于生态系统的研究则可以有效地指导一个区域内多个物种状态评估过程, 并为未来向多物种状态评估过渡提供基础。本研究关注美国东南部濒危的草地物种和生态系统, 该地区草地稀有种和特有种的数量不成比例得高。为了实施基于生态系统的方法, 我们将基于文献和结构化世界咖啡馆研讨会的形式相结合, 以确定物种状态估计的科学需求。我们集中讨论了草地物种和生态系统面临的5类威胁, 并建议将共同存在的威胁作为ESA的规划重点, 这些威胁包括 (1) 栖息地丧失、破碎化和功能连接度中断; (2) 气候变化; (3) 干扰情况变化; (4) 入侵物种; (5) 局部地区的影响。研讨会参与者确定了每种威胁的科学需求和信息需求, 包括数据库可用性、优先研究内容以及建模和测绘需求。根据栖息地和共同威胁对物种进行分组, 可以使物种状态评估过程和全球其它濒危物种保护的规划过程更加有用和高效。我们发现, 将文献综述与结构化讨论相结合, 可以有效地确定支持多个物种状态评估发展所需的科学信息和分析。**【翻译: 胡怡思; 审校: 聂永刚】**

## INTRODUCTION

The U.S. Endangered Species Act (ESA) is one of the world's most powerful and effective biodiversity conservation laws (Goble et al., 2006; Harris et al., 2012). Despite the ESA's success in improving the status of listed species (Greenwald et al., 2019; Schwartz, 2008), the law is not functioning optimally, for example, times for species to gain protection are lengthy (Puckett et al., 2016). Some 1721 U.S. species (as defined under the ESA) are currently listed and the U.S. Fish and Wildlife Service (USFWS) faces a backlog of hundreds of species waiting for listing decisions or designation of critical habitat (USFWS, 2021). Clearly, methods are needed to streamline decision‐making while improving the scientific basis of decisions.

In response to its increasing workload, the USFWS has long sought ways to make decisions and actions under the ESA more efficient. Habitat conservation plans (HCPs), which since 1982 are required for permitting of incidental take of species that occurs during otherwise lawful development, were originally single‐species plans covering small areas. Beginning in the 1990s, multispecies HCPs covering large landscapes were developed to address the needs of many species as well as ecosystems in areas proposed for development (Noss et al., 1997). Similarly, recovery plans evolved in the 1990s from single‐species plans to multispecies plans in many cases. Unfortunately, the track record of multispecies HCPs and recovery plans accomplishing conservation objectives is mixed; several analyses suggest they are of lower quality than single‐species HCPs (Boersma et al., 2001; Clark & Harvey, 2002; Rahn et al., 2006; Mitrovich et al., 2010).

One response to these documented failures would be to abandon multispecies and ecosystem planning and return to purely single‐species plans. Such a response would not resolve the escalating workload within the USFWS, however, and would be inconsistent with the logic and evidence for the effectiveness of ecosystem‐level conservation (Franklin, 1993; Noss, 1996). An ecosystem approach is more cost‐efficient because many species with similar biological requirements and threats can be addressed by the same actions. By protecting ecosystems that are shared by at‐risk species, managers are more likely to account for these species’ individual biological needs, many of which are often poorly known, and protect many other more common species.

A trend toward ecosystem‐level conservation is evident internationally in response to documented limitations of species‐by‐species approaches. In addition to the problem of multiple species not yet being collected or described, the status of 94% of known land plant species has not yet been evaluated under the International Union for Conservation of Nature (IUCN) Red List criteria (Corlett, 2016). Since 2010, under the international Convention on Biological Diversity (CBD), targets for conserving species have helped reduce species loss worldwide, yet no targets have been set for the more cost‐efficient task of conserving ecosystems (Watson et al., 2020). The IUCN recently developed criteria for a Red List of Ecosystems (Keith et al., 2013), which so far have been used to assess the status of more than 2800 ecosystems in 100 countries (Bland et al., 2019). The need remains, however, for a practical integration of species‐ and ecosystem‐level conservation.

Improvements in multispecies and ecosystem‐based conservation require increased involvement of science in decision‐making and implementation of actions. The frequent failure of multispecies or ecosystem plans to achieve conservation objectives can be linked to specific deficiencies in the development and implementation of plans, including flawed reserve design, inadequate identification and monitoring of threats, lack of independent scientific review, political interference, and insufficient funding (Noss et al., 1997; Clark & Harvey, 2002; Rahn et al., 2006; Mitrovich et al., 2010; Henson et al., 2018). These deficiencies can be remedied through such straightforward measures as grouping species by shared habitat requirements, functional traits, or threats (Clark & Harvey, 2002; Kooyman & Rossetto, 2008); improved monitoring of species and ecosystem status and threats (Clark et al., 2002; Hierl et al., 2008; Troyer & Gerber, 2015); and increased funding for prompt listing of imperiled species and rapid designation of critical habitat and development of recovery plans (Taylor et al., 2005; Schwartz, 2008).

We summarized a collaborative project that addresses multispecies and ecosystem planning challenges within the context of the USFWS's recently adopted species status assessment (SSA) process (Smith et al., 2018). An SSA is a biological risk assessment to provide decision‐makers with information on the current and projected future biological status of species listed under the ESA and to aid decisions about which species should be proposed for listing. An SSA is intended to inform all ESA decisions, including listing and delisting, recovery planning, critical habitat designation (Smith et al., 2018), and potentially development of HCPs. Although SSAs, to date, have focused on individual species, we suggest that research concentrating on species that share habitat requirements and threats can efficiently inform multiple single‐species SSAs within a region and lay the foundation for possible future development of multispecies SSAs.

Species status assessments have 3 defined stages (Smith et al., 2018), and an ecosystem‐based approach can contribute substantially to each stage. Stage 1 focuses on the life history and ecology of the species, including its habitat requirements. We explicitly associated federally listed and other at‐risk species with habitats defined by NatureServe ecological system types (Comer et al., 2003) and corresponding types in the U.S. National Vegetation Classification (USNVC, 2019; Appendix S1). Stage 2 assesses the current condition of a species in terms of “habitat, demographics, and distribution” (Smith et al., 2018). Our data on ecological systems and USNVC types allow for the quantification of current habitat condition and extent, historical trends, current land protection status, and key threats. Finally, stage 3 considers the future condition of species. Our ecosystem‐based approach (which includes consideration of shared threats) is efficient; with limited research dollars, scientists can produce information to inform many SSAs simultaneously by focusing on current and potential future conditions of habitats (ecological systems). Future‐condition projections for any species are incomplete without future‐scenario projections on the scale of ecosystems and landscapes. Although some future conditions information, such as demographics and genetics, are necessarily species specific, an ecosystem approach allows for consideration of future habitat conditions of many imperiled species simultaneously. Ultimately, the future viability of every species depends on the extent, configuration, and quality of its habitat (Noss et al., 1997).

**FIGURE 1:**
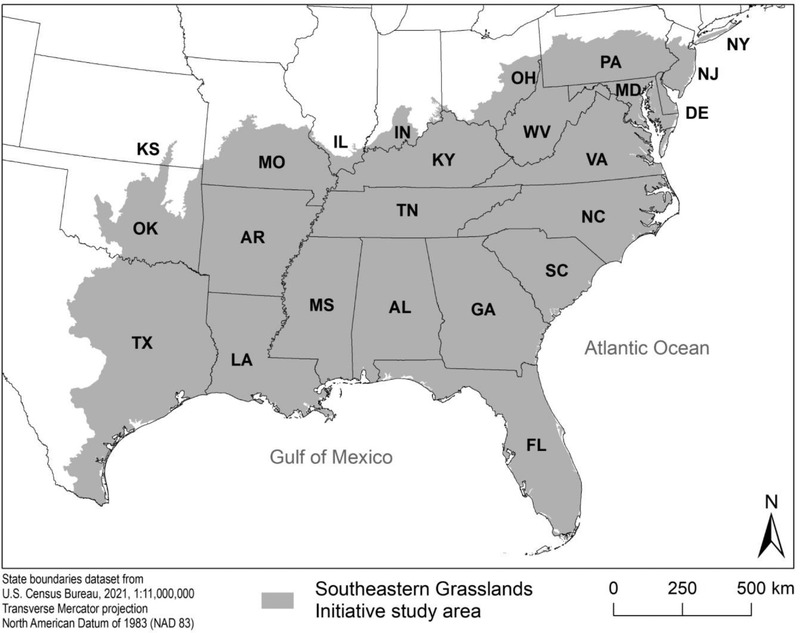
The southeastern U.S. grasslands study region: the biogeographic Southeast as defined by the Southeastern Grasslands Initiative. The northern boundary corresponds to the Wisconsinan and Illinoian glacial boundaries, adjusted slightly by reference to U.S. Environmental Protection Agency (EPA) level III and level IV ecoregions, where there is uncertainty about the glacial boundary. The western boundary is defined by EPA level III and level IV ecoregions to include those ecoregions with a dominant southeastern floristic affinity as opposed to a Great Plains, Tallgrass Prairie, or Madrean affinity. Figure reprinted with permission from Noss et al. (2021)

We used a combined literature‐based and workshop format to identify science needs for SSAs involving grassland species within the unglaciated southeastern United States (Figure 1). In partnership with the Southeastern Grasslands Initiative (SGI), we defined grasslands broadly as ecosystems within which plant cover or biomass, and usually species richness, are concentrated in a graminoid‐dominated herbaceous layer (Frost, 1998; Noss, 2013). This definition encompasses prairies, savannas, open woodlands, glades, barrens, balds, meadows, marshes, graminoid‐dominated fens and bogs, and dunes, but excludes communities, such as outcrops, domes, and beaches with very sparse vegetative cover. Our definition is considerably more inclusive than some well‐accepted global definitions of grasslands, such as in the International Vegetation Classification (Dixon et al., 2014; Faber‐Langendoen et al., 2020). As determined by vote of an ad hoc vegetation committee of the SGI, some 118 ecological systems in the NatureServe classification (Comer et al., 2003) met our definition (Appendix S1).

This region and set of ecosystems were selected for our case study because of their extraordinary species richness and endemism due to long‐term relative climatic stability, diverse geology and soils, and other factors (Noss, 2013; Noss et al., 2015). The region also faces extreme threats. Losses of southeastern grasslands since EuroAmerican settlement are estimated at 90% overall, with many types approaching 100% (Noss et al., 1995; Comer et al., 2020). Accordingly, more than 600 ESA‐listed and other at‐risk taxa are concentrated in southeastern grasslands, currently known from 94 of the 118 ecological systems (Appendix S2).

## METHODS

Recognized subject‐matter experts, including grassland researchers, managers, and administrators from federal and state agencies, nongovernmental organizations, and universities (Appendix S3), held a regional SSA workshop in January 2020 in Morrison, Tennessee, to identify science needs for the conservation of southeastern grassland ecosystems and species. The workshop, organized by SGI and the U.S. Geological Survey (USGS), focused on identifying specific types of scientific information and research needed to support the USFWS and state agencies in the development of SSAs for grassland species listed under the ESA or under current or potential consideration for listing. The participants were selected for their expertise in southeastern grassland ecology and to represent key stakeholders, especially state agencies tasked with grassland conservation.

Thirty‐nine workshop participants were asked to evaluate a literature review on southeastern grassland ecology and conservation, prepared by R.N. and circulated as a detailed outline prior to the workshop, as well as literature‐based information presented by plenary speakers. Participants were then asked to use their knowledge, expertise, and additional literature to revise and expand the outline into the basis of a USGS report (Noss et al., 2021). Three sections of the outline were drawn primarily from literature synthesis and were only slightly revised by the workshop process: an introduction, overview of an ecosystem‐based approach to SSAs, and a summary of threats to grassland ecosystems. The last section of the outline contained general points on science needs for proposed discussion in the workshop. This section was greatly expanded by the workshop participants as they identified, discussed, and documented dozens of research needs and knowledge gaps to support grassland conservation and the SSA process.

The workshop format included several plenary presentations followed by 2 half days of breakout group discussions and, finally, group reports and whole‐group discussion and revision of the report outline. Workshop participants were assigned randomly to 5 groups for breakout sessions, resulting in an average of 8 participants per group. Breakout sessions applied a modified World Café approach, which is a structured conversational process for knowledge sharing in which breakout groups discuss topics separately. Groups switch tables periodically and are introduced to previous discussions by a table host, who facilitates discussion and takes notes (World Café, 2020). Thus, by cycling through World Café tables, each group and every participant have an opportunity to discuss each topic. Table hosts compiled detailed notes on the comments made by each of the 5 groups. Toward the end of the workshop, table hosts presented summaries of these notes to the entire body of workshop participants for additional comments and clarification, and then submitted their final notes to R.N. These 5 documents (finalized notes from each table host) were used to revise the outline for the USGS report (Noss et al., 2021) to encompass the full range of participants’ opinions, concerns, and desires concerning science needs for southeastern grassland conservation. Thus, the World Café approach provided a valuable complement to the literature synthesis by capturing grassland managers’ on‐the‐ground experiences, observations, and priorities, a necessary component of any translational ecology approach (Enquist et al., 2017).

The workshop steering committee determined in advance to focus discussions on science needs related to major threats that affect multiple species and grassland ecosystems in the region. This emphasis on threats is consistent with published recommendations to make threats a primary focus of multispecies planning (Clark et al., 2002), group species by shared threats (Clark & Harvey, 2002), and incorporate threat monitoring into plans (Troyer & Gerber, 2015). This focus on threats was also informed by guidelines for the SSA process, which specify that the future condition of species be assessed based on “future plausible scenarios of stressors and conservation efforts” (Smith et al., 2018).

Five categories of threats were selected for discussion. Four applied to all grassland communities and species within the region, and can be considered broadly shared threats, and the fifth applied to more spatially restricted situations: (1) habitat loss, fragmentation, and disruption of functional connectivity; (2) climate change; (3) altered disturbance regimes; (4) invasive species; and (5) localized impacts. For each threat, participants focused on science and information needs, such as database availability, field survey and research priorities, modeling and mapping needs, and conservation and management priorities.

## RESULTS

The ultimate threats to grasslands and other southeastern ecosystems are urban expansion and other land‐use changes (Terando et al., 2014). Unable to address these ultimate threats, resource managers can focus on proximal threats. Results from workshop discussions on the identified threats results represent problems, hypotheses, and questions that can be addressed by future research and monitoring. Ideally, these threats and their repercussions will be considered explicitly during the development of individual SSAs.

### Habitat loss, fragmentation, and disruption of functional connectivity

The loss and fragmentation of natural habitat is generally accepted as the leading proximal threat to biodiversity (Haddad et al., 2015; Chase et al., 2020). Fragmentation may be more problematic for some types of ecosystems and species than others. Small patch communities, such as many edaphic grasslands, are inherently small and isolated. Their species are adapted to this condition, albeit changes in the surrounding matrix may create deleterious edge effects. The value of small patches for biodiversity, especially from the criteria of complementarity and representativeness, is high but underappreciated (Wintle et al., 2018). Grasslands and other communities that are naturally matrix or large‐patch communities may experience greater negative impacts of anthropogenic fragmentation, especially for area‐sensitive animal species or any species sensitive to edge effects. Loss of functional connectivity, measured by successful movements and gene flow (Tischendorf & Fahrig, 2000), can negatively affect species sensitive to fragmentation.

The following science needs related to fragmentation and connectivity were noted as most critical for SSA: (1) Identify, through remote sensing, mapping, and modeling, the southeastern grassland ecosystems that are most threatened by land‐use change. (2) Improve maps of historic and current distributions of all southeastern grassland types and determine extents and rates of habitat loss or conversion, which can guide prioritization of ecosystems and sites for conservation and restoration. (3) Determine the minimum grassland patch size needed to support viable populations of at‐risk grassland‐dependent and fragmentation‐sensitive species. (4) Improve understanding of how fragmentation may disrupt plant–animal interactions, including pollination, obligate herbivory or parasitism, and animal‐mediated seed dispersal. (5) Evaluate the feasibility of restoring grasslands that have been lost to agricultural conversion and of potential grassland dispersal corridors, such as powerlines and other rights‐of‐way.

### Climate change

Climate change is a profound but complicated threat to grasslands and their native species. Forests have received considerably more attention with respect to potential climate impacts and various mitigation and adaptation options. Moreover, planting trees (afforestation) to capture carbon has become a major threat to many types of grasslands worldwide (Veldman et al., 2015). Ironically, grasslands may be a more secure carbon sink than forests during a time of increasing temperature, drought, and wildfire because they store most of their carbon securely belowground (Dass et al., 2018).

Documented trends and climate projections in the Southeast include increases in average temperatures, numbers of warm nights, and length of the frost‐free season (USGCRP, 2017, 2018). Evapotranspiration increases with temperature, potentially reducing available water to plants if precipitation is insufficient. Oceans are warming and sea level is rising, causing more frequent inundation of coastal ecosystems and more intense hurricanes. Extreme rainfall events are projected to become more frequent and severe, although the potential changes to future average seasonal precipitation are less certain because the frequency of dry days may increase. As temperatures rise, drought is expected to become more frequent and severe. Climate change also is projected to increase the frequency and intensity of wildfires and place greater constraints on controlled burning for ecosystem management (USGCRP, 2017, 2018; Kupfer et al., 2020). Despite high confidence in these predicted trends, the threats posed by climate change to southeastern grasslands and the options for adaptation remain poorly understood (Cartwright & Wolfe, 2016).

The following climate‐related science needs stand out as most urgent for SSA. (1) Identify grassland species that are unable to shift spatially in response to climate change due to restrictions to high elevations, specific soil types, geologic formations, hydrologic settings (e.g., seepages), or physical processes. These species occur in grasslands associated with riverscour, mountaintops, glades, seeps, fens, barrens, coastal dunes, and marl prairie, among others (Noss, 2013). (2) Dispersal distances and rates—including abilities to move between habitat patches—are vital considerations for climate adaptation but are unknown for many species. (3) For species of conservation concern, identify the physiological thresholds that determine population viability. Examples of potential threshold phenomena include germination requirements and tolerances to cold, heat, inundation, salt, and drought. Some life‐history stages may be particularly sensitive (e.g., seedling establishment may be a stronger limiting factor than germination [Walck et al., 2011]). (4) Observational studies are needed on potential phenophase mismatch (e.g., for the timing of bud‐burst to shift asynchronously with the timing of pollinator visitation or the arrival of migratory birds asynchronous with insect emergence). (5) Climate change is predicted to increase lightning activity by perhaps 50% during this century (Romps et al., 2014). Will increased lightning result in more wildfires, potentially favoring grassland over forest, or will any increase in fire be counteracted by increased fire suppression? (6) Identify and map potential climatic microrefugia (Dobrowski, 2011) for grassland species throughout the Southeast. Potential microrefugia can be identified through fine‐scale topo‐climatic modeling, geological data, and inferences from paleoecology and phylogenetics. Even if putative microrefugia turn out to provide only temporary relief for species at risk (Brown et al., 2020; Morelli et al., 2020), this short‐term persistence may buy time for other conservation actions, such as ex situ conservation. (7) Should some grassland systems, sites, or species be allowed to disappear? No other option may exist for those threatened by sea‐level rise and other such immediate, irreversible, and essentially permanent threats (see 1 above). What methods should be used to identify sites, ecosystems, and species that fall into this category?

### Altered disturbance regimes

In relatively rainy regions, such as the southeastern United States, fire can override the influence of precipitation in determining the distribution and abundance of vegetation types (Bond et al., 2005). Many southeastern grasslands are disturbance dependent. In addition to fire, these grasslands may be maintained by floods, herbivory, windstorms, drought, and other disturbances (Noss, 2013). Because species have adapted to specific disturbance regimes (Keeley et al., 2011), disturbance alteration may produce conditions outside the evolutionary experience of native species, putting them at potential risk of extinction. Thus, changing disturbance regimes, often related to climate or land‐use change, constitute a major threat to southeastern grasslands.

Critical research questions and science needs for consideration in SSAs include the following. (1) Information on fire regimes in the Southeast other than for longleaf pine (*Pinus palustris*) ecosystems is limited. There is the need for high‐resolution data from dendrochronology, historical surveys, and other approaches to determine the evolutionary (lightning‐ignited) fire regimes for grassland types throughout the region, in terms of frequency, seasonality, severity, patch size, heterogeneity, and other factors. (2) What is the range of variability in disturbance regimes that grassland species experienced during their evolutionary histories? Past conditions, in some cases extending back millions of years, are informative of the range of variability that may be tolerated by grassland species in the future. Altering that range of variability in either direction could potentially increase extinction risk. (3) How much unburned habitat is required within a prescribed burn unit to provide adequate refugia for insects and other species vulnerable to fire? (4) How can landscape context (e.g., the matrix surrounding remnant grasslands) be better considered in disturbance management? For example, smoke management is difficult for grassland patches within an urban matrix or adjacent to major highways. (5) To what extent are grassland systems maintained or shaped by hydrology and ecosystem engineers, such as beaver (*Castor canadensis*) for wet meadows, bogs, fens, and canebrakes? How does the hydrological regime affect woody plant encroachment into grasslands, and vice versa? (6) What are the ecological roles and effects of grazing on southeastern grassland plants and animals? Grasslands and species that may be grazing dependent more than fire dependent need to be identified and mapped. (7) How can managed disturbance best be applied to small remnant grasslands? An important caution is that large landscape processes do not necessarily apply to small remnants (Buckles & Harmon‐Threatt, 2019).

### Invasive species

Invasions by non‐native species and weedy native species constitute a fundamental threat to many natural communities and native species, one that may intensify with climate change. In addition to intertrophic actions (i.e., predation, parasitism, and disease), competition between non‐native and rare native plants has been documented in the southeastern United States (e.g., Walck et al., 1999). Other common impacts of invasive plants occur through alteration of natural disturbance regimes, especially fire, or through changes in nutrient levels, for example, with invasion by a nitrogen‐fixing legume.

Important concerns, questions, and research needs include the following. (1) What influences susceptibility of a community to invasion? Does increased nitrogen from fertilizer or livestock adjacent to grassland remnants increase invasion potential? Are more alkaline, limestone‐derived soils more prone to invasion than acidic soils? (2) Invasive species usually do not establish as effectively in intact or old‐growth ecosystems as in degraded ones (Lonsdale, 1999). Better definitions and reference concepts for old‐growth grasslands (Bond, 2016) in the Southeast are needed. Research is needed to identify ecological factors that differ between intact and degraded grasslands that influence invasion susceptibility. (3) How does chemical herbicide treatment of non‐native species affect rare native plants? (4) A DNA barcode database is needed for rusts and smuts that can be used in the early detection of infections in grassland plants.

### Localized impacts

Finally, the workgroups considered information needs associated with localized or subregional impacts, such as sea‐level rise, as well as other issues, sometimes species specific, that did not easily fit within the above threat categories. Because these issues are, by definition, specific to particular places, their level of general interest is lower than the 4 categories addressed above, so we do not summarize them here (but see Noss et al., 2021).

## DISCUSSION

We sought to apply lessons from 3 decades of experience in multispecies and ecosystem‐level planning to the SSA process under the ESA, while illuminating global principles for addressing the conservation needs of numerous imperiled species in any region cost‐efficiently. A major conclusion is that, although SSAs currently focus on individual species, a species‐by‐species approach to SSAs for southeastern U.S. grasslands would be extremely cumbersome due to the immense number of grassland‐associated at‐risk species (Appendix S2). Instead, multispecies research and planning is generally more efficient and practical but must be scientifically rigorous and adaptable over time (Noss et al., 1997). We grouped species by shared ecosystem types (NatureServe's ecological systems and hierarchical USNVC types [Appendices S1 and S2]) and identified shared threats (cf. Clark & Harvey, 2002) as the basis for workshop discussions and identification of science needs. Further research could cluster species by trait‐based functional groups (Kooyman & Rossetto, 2008) or other ecologically meaningful criteria for more detailed evaluations of species conservation needs.

We found a combination of pre and postworkshop literature review and structured workshop discussions effective for identifying the types of scientific information and analysis needed to support the development of SSAs for species currently listed under the ESA or under consideration for listing. Our case study of southeastern grassland ecosystems and species is exemplary of biodiversity hotspots more generally, due to the extraordinarily high irreplaceability (e.g., endemism) and vulnerability (e.g., urban expansion) within this region. With so many species and ecosystems at risk, it is imperative that the SSA process be efficient and scientifically defensible, while yielding positive conservation outcomes.

Biologists now recognize that addressing the conservation needs of imperiled species individually in regions with large numbers of such species is cumbersome, if not impossible. For example, ter Steege et al. (2015) estimated extinction risk for more than 15,000 Amazonian tree species––almost as many as the total number of plant species evaluated globally by the IUCN over a half century––and determined that 36–57% probably qualify for threatened status under IUCN Red List criteria. Imagine trying to develop individual SSAs or recovery plans for so many species in a single region. Identifying, mapping, and developing conservation strategies for the ecosystems upon which multiple imperiled species depend would be much more practical and responsive to urgent conservation needs. The surge of activity in developing red lists of ecosystems in many countries (e.g., Berg et al., 2014; Bland et al., 2019; Watson et al., 2020) signals increased interest in conservation at the ecosystem level despite perceived challenges, such as lack of consistent classification or assessment protocols (Boitani et al., 2015). Such challenges are subsiding as both the IUCN global ecosystem typology (Keith et al., 2020) and the International Vegetation Classification (Faber‐Langendoen et al., 2020) are further refined and assessment methods are better developed (Keith et al., 2015; Comer et al., 2020). The linkage of species‐level to ecosystem‐level conservation needs will become clearer as more regions and countries develop lists of at‐risk taxa associated with specific ecosystem types, as we have done here (Appendix S2).

Our approach is not without challenges and limitations. Synthesizing and summarizing the enormous volume of highly detailed notes from breakout‐group discussions into a coherent written report was difficult. Results from group discussions may vary depending on the mix of participants and may not represent perspectives from experts who were unable to attend. Finally, workshop participants repeatedly noted that generalizations about how to apply the SSA process for southeastern grassland species can only be taken so far. The broad case‐study region encompasses tremendous heterogeneity in climate, landform, geology, soils, hydrology, vegetation, flora, and fauna. Recognizing and understanding local ecological conditions and history is vital for conservation planning.

A multispecies and ecosystem‐based approach to SSAs also faces challenges from a policy perspective. As noted earlier, current USFWS policy does not allow for multispecies SSAs. Recent petitions to list multiple species (e.g., a 2010 petition to protect 404 aquatic and wetland species in the southeastern United States [Center for Biological Diversity, 2010]) are still awaiting decisions for most of the petitioned species, and the agency has since prohibited multispecies petitions through regulation (Federal Register, 2016). Nevertheless, multispecies and ecosystem‐based research and planning during the 3 stages of the SSA process (Smith et al., 2018) can lead to the improvement and increased cost‐efficiency of multiple single‐species SSAs within the existing policy framework, while laying the groundwork for policy evolution toward a stronger ecosystem approach in the future.

## Supporting information


**Appendix S1. Southeastern Grasslands Classification Hierarchy**: List of Ecological Systems and their relation to USNVC types at Group level.

Appendix S2. Federally listed Threatened and Endangered Species and NatureServe Globally Critically Imperiled (G1), Imperiled (G1), and Vulnerable (G3) species, plus subspecies and varieties (T1, T2, T3) documented by NatureServe to be associated with grassland ecological systems in the southeastern U.S. (see Appendix S1); 94 of 118 ecological systems are known to have these at‐risk species associated with them.

Appendix S3. Participants in the workshop in Morrison, Tennessee, January 22‐23, 2020, “Assessing the Science Needs of Southeastern Grassland Species of Conservation Concern.”
