# Supplementary material for: Improving species status assessments under the U.S. Endangered Species Act and implications for multispecies conservation challenges worldwide
Source: Conserv Biol. 2021 Jun 28;35(6):1715–24. doi: 10.1111/cobi.13777 (PMC9292301; doi:10.1111/cobi.13777)
Supplement: Supplementary file 3 — Appendix S3. Participants in the workshop in Morrison, Tennessee, January 22‐23, 2020, “Assessing the Science Needs of Southeastern Grassland Species of Conservation Concern.” [file COBI-35-1715-s003.docx]

Appendix S3. Participants in the workshop in Morrison, Tennessee, January 22-23, 2020, “Assessing the Science Needs of Southeastern Grassland Species of Conservation Concern.” Steering committee members are marked with *.

Daniel Adams, U.S. Fish and Wildlife Service

Matthew Albrecht, Missouri Botanical Garden

Jessi Allen, Atlanta Botanical Garden

Ryan Boyles, U.S. Geological Survey

Kyle Brazil, American Bird Conservancy

Cooper Breeden, Southeastern Grasslands Initiative

Geoff Call, U.S. Fish and Wildlife Service*

Jennifer Cartwright, U.S. Geological Survey*

Bashira Chowdhury, Auburn University

Emily Coffey, Atlanta Botanical Garden

Patrick Comer, NatureServe

Todd Crabtree, Tennessee Department of Environment and Conservation

Adam Dattilo, Tennessee Valley Authority

Chris Doffitt, Louisiana Department of Wildlife and Fisheries

Sam Droege, U.S. Geological Survey

Caitlin Elam, Tennessee Department of Environment and Conservation

Gregg Elliott, Southeastern Grasslands Initiative*

Dwayne Estes, Southeastern Grasslands Initiative*GIS

David Hanni, Tennessee Department of Environment and Conservation

JoVonn Hill, Mississippi State University

Chuck Hunter, U.S. Fish and Wildlife Service*

Zach Irick, Southeastern Grasslands Initiative

Todd Jones-Farrand, U.S. Fish and Wildlife Service

Wesley Knapp, North Carolina Natural Heritage Program

David Lincicome, Tennessee Department of Environment and Conservation

Tara Littlefield, Office of Kentucky Nature Preserves

Kathy Marquart, Southeastern Grasslands Initiative

Mike Marshall, Texas A&M University and U.S. Fish and Wildlife Service*

Reed Noss, Florida Institute for Conservation Science*

Milo Pyne, NatureServe, retired

Nicole Rankin, U.S. Fish and Wildlife Service

Al Schotz, Auburn University

Guenter Schuster, Eastern Kentucky University

Jason Singhurst, Texas Parks and Wildlife Department

Christopher Tracey, Pennsylvania Natural Heritage Program

James Vanderhorst, West Virginia Natural Heritage Program

Jeffrey Walck, Middle Tennessee State University

Scott Wiggers, U.S. Fish and Wildlife Service

Theo Witsell, Southeastern Grasslands Initiative*
